# Cross-sectional study of psychiatric comorbidities in patients with atopic dermatitis and nonatopic eczema, urticaria, and psoriasis

**DOI:** 10.2147/NDT.S191509

**Published:** 2019-05-28

**Authors:** Hye-Jin Ahn, Min Kyung Shin, Jong-Kil Seo, Su Jin Jeong, Ah Rang Cho, Sun-Hee Choi, Bark-Lynn Lew

**Affiliations:** 1 Department of Dermatology, College of Medicine, Kyung Hee University, Seoul, Korea; 2 Medical Science Research Institute, Kyung Hee University Medical Center, Seoul, Korea; 3 Department of Psychiatry, Kyung Hee University Hospital at Gangdong, Kyung Hee University School of Medicine, Seoul, Korea; 4 Department of Pediatrics, Kyung Hee University Hospital at Gangdong, Kyung Hee University School of Medicine, Seoul, Korea; 5 Department of Dermatology, Kyung Hee University Hospital at Gangdong, Kyung Hee University School of Medicine, Seoul, Korea

**Keywords:** atopic dermatitis, Korean National Health Insurance Research Database, mental illness

## Abstract

**Background:** Recent data suggest depression has been linked to chronic skin diseases, including atopic dermatitis (AD), urticaria, and psoriasis. This study compared mental illnesses in patients with AD with those of patients with nonatopic eczema, urticaria, and psoriasis in Korea.

**Methods:** A cross-sectional study design was used, analyzing data from the 2015 Korean National Health Insurance Research Database, a survey of 42,641 AD and 139,486 non-AD (nonatopic eczema, urticaria, and psoriasis) patients (103,938 males, 78,189 females) classified by age: infant, aged 0–3 years; early childhood, aged 4–8 years; late childhood, aged 9–12 years; adolescent, aged 13–18 years; adult, aged 19–64 years; elderly, aged above 65 years. Multiple logistic regression analysis was performed, and the odds ratio (OR) of various mental illnesses – attention-deficit/hyperactivity disorder (ADHD), autism spectrum disorder (ASD), conduct disorder, depression, anxiety, suicidal ideation, schizophrenia, and sleep disorder – were calculated for patients with and without AD.

**Results:** The incidence of depression was not significantly different between AD and non-AD patients. Severe AD showed a high OR of depression (moderate AD OR=1.75; severe AD OR=3.15, *P*<0.0001). Patients with AD had significantly higher incidence of ADHD (OR=1.48; 95% CI=1.27–1.72), ASD (OR=1.54; 95% CI=1.19–1.99), and conduct disorder (OR=2.88; 95% CI=1.52–5.45).

**Conclusion:** Patients with AD were not found to have higher incidence of depression than non-AD patients. However, severe AD patients were determined to have a significantly higher incidence of depression. Therefore, the severity of dermatitis is thought to contribute to depression. Mental illnesses found to be significantly higher in AD patients were ADHD, ASD, and conduct disorder.

## Introduction

Atopic dermatitis (AD), a common chronic inflammatory skin disease that may persist into adulthood, is associated with intense pruritus, high rates of sleep disturbance, and poor quality-of-life.^1^,^2^ The prevalence of AD is 15–20% among children, and 1–3% among adults worldwide.^3^ Psychological distress is one of the common comorbidities linked to AD, and could negatively affect quality-of-life.^4^ Previous large-scale population studies have found an association between AD and mental illnesses.^5^–^8^ Children with AD in the US had significantly higher prevalence of attention-deficit/hyperactivity disorder (ADHD; odds ratio [OR]=1.87; 95% confidence interval [CI]=1.54–2.27), depression (OR=1.81; 95% CI=1.33–2.46), anxiety (OR=1.77; 95% CI=1.36–2.29), conduct disorder (OR=1.87; 95% CI=1.46–2.39), and autism (OR=3.04; 95% CI=2.13–4.34) than those without AD.^5^ Cheng et al^6^ found that Taiwanese patients with AD had an elevated risk of developing major depression (MDD) (hazard ratio [HR]=6.56; 95% CI=3.64–11.84), depressive disorders (HR=5.44; 95% CI=3.99–7.44), and anxiety disorders (HR=3.57; 95% CI=2.55–4.98). In Korea, Kim et al^7^ found that depressive symptoms were significantly higher in AD patients than in matched controls. A recent study of adolescents from Korea found slightly increased risks of suicidal ideation, planning, and attempts in patients with AD.^8^ However, these previous studies investigated differences between AD and normal controls. A wide range of dermatologic disorders, such as AD, psoriasis, and chronic urticaria, have been reported to be associated with depression.^9^ The objective of our study was to perform a comprehensive comparison of mental illnesses in all age groups of patients with AD and patients with other chronic skin disease through a large population-based survey in Korea.

## Materials and methods

### Data source

A cross-sectional study design was used to analyze the Korean National Health Insurance Research Database for 2015, a survey of 42,641 AD and 139,486 non-AD (nonatopic eczema, urticaria, and psoriasis) patients. Medical records were analyzed according to the codes of the International Statistical Classification of Diseases (ICD-10). We also investigated data from 2002 to 2014 for past AD (L20.9) diagnostic records. The study was approved by the Institutional Review Board of Kyung Hee University Hospital (KMC IRB 2016-05-406).

### Inclusion criteria of patients and classification of data

We classified patients by age: infant, 0–3 years; early childhood, 4–8 years; late childhood, 9–12 years; adolescence, 13–18 years; adulthood, 19–64 years; elderly, above 65 years.

Patients with non-AD were considered as those with nonatopic eczema, urticaria (L50.9), or psoriasis (L40.9). Nonatopic eczema included seborrheic dermatitis (L21.9), irritant contact dermatitis (L24.9), and allergic contact dermatitis (L23.9).

It was difficult to classify the severity of AD using severity scores. Therefore, we divided the group as follows: patients with mild AD included only those receiving topical treatment, patients with moderate AD were those treated with antihistamines, and severe AD patients were defined as those treated with systemic immunosuppressive therapy. Patients were considered to have a history of AD if they were diagnosed with AD at least once between 2002 to 2014.

Mental illnesses included ADHD (F90.0), ASD (autism spectrum disorder) (F84.0), conduct disorder (F91.9), depression (F32.2, F32.8, F32.9), anxiety (F41.9), suicidal ideation (R45.8), schizophrenia (F20.9), and sleep disorder (G47.9).

### Statistical analysis

Multiple logistic regression models were created for each mental illness to control for known confounders that might influence the relationship between AD and each mental illness. A *P*-value of less than 0.05 was considered statistically significant. All data processing and statistical analyses were performed with IBM SPSS Statistics version 21 (IBM) and SAS version 9.2 (SAS Institute, Cary, NC, USA).

## Results

Overall, 182,127 patients were included in our study: 31,471 infants, 25,227 in early childhood, 14,677 in late childhood, 29,526 adolescents, 67,878 adults, and 13,348 elderly. There were 42,641 patients with AD, 71,699 with nonatopic eczema, 62,464 with urticaria, and 5,323 with psoriasis.

### Prevalence rate of mental illnesses in AD and non-AD patients

We analyzed the frequency of each psychiatric disease in AD and non-AD patients. We calculated that the prevalence of depression in AD patients was 2.47% (Table 1).

**Table 1:** Prevalence rate of mental illnesses in AD and non-AD patients by age

Mental illness		Frequency (prevalence rate [%])							
Total all patients	Infant	Early childhood	Late childhood	Total childhood	Adolescent	Adult	Elderly
**Total number of patients**	**AD**	**42,641**	**6,219**	**6,132**	**4,217**	**10,349**	**8,339**	**14,874**	**2,860**
**Non-AD**	**139,486**	**25,252**	**19,095**	**10,460**	**29,555**	**21,187**	**53,004**	**10,488**
**Attention-deficit/hyperactivity disorder**	**AD**	240 (0.56)	–	45 (0.73)	74 (1.75)	119 (1.15)	97 (1.16)	24 (0.16)	–
**Non-AD**	655 (0.47)	12 (0.05)	171 (0.90)	182 (1.74)	353 (1.19)	209 (0.99)	78 (0.15)	3 (0.03)
**Autism spectrum disorder**	**AD**	88 (0.21)	10 (0.16)	19 (0.31)	16 (0.38)	35 (0.34)	24 (0.29)	19 (0.13)	–
**Non-AD**	212 (0.15)	36 (0.14)	59 (0.31)	22 (0.21)	81 (0.27)	49 (0.23)	45 (0.08)	1 (0.01)
**Conduct disorder**	**AD**	17 (0.04)	1 (0.02)	1 (0.02)	2 (0.05)	3 (0.03)	10 (0.12)	3 (0.02)	–
**Non-AD**	25 (0.018)	–	6 (0.03)	2 (0.02)	8 (0.03)	13 (0.06)	2 (0.00)	2 (0.02)
**Depression**	**AD**	1,052 (2.47)	–	8 (0.13)	22 (0.52)	30 (0.29)	105 (1.26)	543 (3.65)	374 (13.08)
**Non-AD**	4,728 (3.39)	2 (0.01)	37 (0.19)	79 (0.76)	116 (0.39)	364 (1.72)	2,499 (4.71)	1,747 (16.66)
**Anxiety**	**AD**	1,448 (3.40)	5 (0.08)	19 (0.31)	24 (0.57)	43 (0.42)	109 (1.31)	758 (5.10)	533 (18.64)
**Non-AD**	6,510 (4.67)	43 (0.17)	80 (0.42)	78 (0.75)	158 (0.53)	420 (1.98)	3,482 (6.57)	2,407 (22.95)
**Suicidal ideation**	**AD**	176 (0.41)	–	2 (0.03)	2 (0.05)	4 (0.04)	14 (0.17)	80 (0.54)	61 (2.13)
**Non-AD**	832 (0.60)	–	12 (0.06)	3 (0.03)	15 (0.05)	35 (0.17)	388 (0.73)	292 (2.78)
**Schizophrenia**	**AD**	130 (0.30)	–	2 (0.03)	–	2 (0.02)	11 (0.13)	101 (0.68)	16 (0.56)
**Non-AD**	489 (0.35)	–	7 (0.04)	16 (0.15)	23 (0.08)	44 (0.21)	341 (0.64)	81 (0.77)
**Sleep disorder**	**AD**	938 (2.20)	3 (0.05)	3 (0.05)	6 (0.14)	9 (0.09)	24 (0.29)	507 (3.41)	395 (13.81)
**Non-AD**	4,523 (3.24)	58 (0.23)	27 (0.14)	23 (0.22)	50 (0.17)	111 (0.52)	2,504 (4.72)	1,800 (17.16)
**Allergic conjunctivitis**	**AD**	6,195 (14.53)	602 (9.68)	1,363 (22.23)	915 (21.70)	2,278 (22.01)	1,192 (14.29)	1,814 (12.20)	309 (10.80)
**Non-AD**	25,958 (18.61)	3,923 (15.61)	5,572 (29.18)	2,835 (27.10)	8,407 (28.45)	3,831 (18.08)	8,484 (16.00)	1,313 (12.52)
**Allergic rhinitis**	**AD**	20,863 (48.93)	4,308 (69.27)	4,307 (70.24)	2,437 (57.79)	6,744 (65.17)	3,580 (42.93)	5,162 (34.70)	1,069 (37.38)
**Non-AD**	80,866 (57.97)	20,539 (81.34)	15,107 (79.11)	6,748 (64.51)	21,855 (73.95)	10,544 (49.77)	22,854 (43.12)	5,074 (48.38)
**Asthma**	**AD**	6,823 (16.00)	2,540 (40.84)	1,856 (30.27)	621 (14.73)	2,477 (23.93)	522 (6.26)	902 (6.06)	382 (13.36)
**Non-AD**	29,504 (21.15)	12,347 (48.90)	7,323 (38.35)	1,965 (18.79)	9,288 (31.43)	1,743 (8.23)	4,257 (8.03)	1,869 (17.82)
**Previous AD history**	**AD**	33,559 (78.70)	2,615 (42.05)	5,262 (85.81)	3,964 (94.00)	9,226 (89.15)	7,797 (93.50)	11,993 (80.63)	1,928 (67.41)
**Non-AD**	–	–	–	–	–	–	–	–

### Mental illnesses according to AD

In the multiple logistic regression models, there was no significant difference in the prevalence of depression between AD and non-AD patients (Table 2). Patients with AD demonstrated increased prevalence of ADHD (OR=1.48; 95% CI=1.27–1.72), ASD (OR=1.54; 95% CI=1.19–1.99), and conduct disorder (OR=2.88; 95% CI=1.52–5.45), and lower prevalence of anxiety (OR=0.91; 95% CI=0.85–0.97) and sleep disorder (OR=0.85; 95% CI=0.79–0.92) than patients with non-AD. There was no significant difference in schizophrenia or suicidal ideation.

**Table 2:** Multiple logistic regression analysis of mental illnesses and atopic dermatitis (AD) in total patients

Mental illness	AD	
Odds (95% CI)^†^	*P*-value
**Attention-deficit/hyperactivity disorder^a^**	1.48 (1.27~1.72)	<0.0001
**Autism spectrum disorder**^a^	1.54 (1.19~1.99)	0.0010
**Conduct disorder^a^**	2.88 (1.52~5.45)	0.0011
**Depression**	0.94 (0.88~1.01)	0.0991
**Anxiety**	0.91 (0.85~0.97)	0.0036
**Suicidal ideation**	0.90 (0.76~1.06)	0.2143
**Schizophrenia**	1.08 (0.88~1.31)	0.4777
**Sleep disorder**	0.85 (0.79~0.92)	<0.0001

**Notes:** *Adjusted by age, gender, economic status, severity of atopic dermatitis, AD history, concomittent allergic disease. †Odds ratio calculated vs non-AD controls. ^a^Means are significantly different.

### Mental illnesses in AD, urticaria, and psoriasis compared to non-AD eczema

As shown in Table 3, there was no significant difference in depression between urticaria and AD patients and nonatopic eczema patients. However, patients with psoriasis showed a higher prevalence of depression (OR=1.38; 95% CI=1.23–1.56). While the results showed that the prevalence of ADHD (OR=1.25; 95% CI=1.06–1.48) and conduct disorder (OR=2.74; 95% CI=1.30–5.78) increased in patients with AD, there was no significant difference in ASD. Patients with urticaria demonstrated increased prevalence of anxiety (OR=1.10; 95% CI=1.04–1.16), suicidal ideation (OR=1.33; 95% CI=1.15–1.53), and sleep disorder (OR=1.18; 95% CI=1.11–1.26). Patients with psoriasis showed an increased prevalence of anxiety (OR=1.16; 95% CI=1.03–1.30), sleep disorder (OR=1.29; 95% CI=1.14–1.47), and depression. AD patients did not show significantly different prevalence rates of anxiety, suicidal ideation, or sleep disorder.

**Table 3:** Multiple logistic regression analysis of mental illnesses in atopic dermatitis (AD) compared to non-AD eczema, urticaria, and psoriasis

Mental illness	Skin disease	Odds (95% CI)^†^	*P*-value
**Attention-deficit/hyperactivity disorder**	AD	1.25 (1.06~1.48)	0.0091
	Urticaria	0.68 (0.58~0.80)	<0.0001
Psoriasis	0.97 (0.64~1.49)	0.9023
**Autism spectrum disorder**	AD	1.24 (0.94~1.64)	0.1312
	Urticaria	0.61 (0.46~0.81)	0.0007
Psoriasis	0.63 (0.26~1.57)	0.3226
**Conduct disorder**	AD	2.74 (1.30~5.78)	0.0084
	Urticaria	0.75 (0.32~1.75)	0.4980
Psoriasis	3.30 (0.91~11.99)	0.0696
**Depression**	AD	0.96 (0.89~1.04)	0.3401
	Urticaria	1.01 (0.94~1.07)	0.8664
Psoriasis	1.38 (1.23~1.56)	<0.0001
**Anxiety**	AD	0.96 (0.89~1.03)	0.2056
	Urticaria	1.10 (1.04~1.16)	0.0012
Psoriasis	1.16 (1.03~1.30)	0.0109
**Suicidal ideation**	AD	1.03 (0.86~1.24)	0.7521
	Urticaria	1.33 (1.15~1.53)	0.0001
Psoriasis	1.01 (0.74~1.39)	0.9421
**Schizophrenia**	AD	1.02 (0.82~1.26)	0.8959
	Urticaria	0.83 (0.69~1.00)	0.0522
Psoriasis	1.35 (0.96~1.89)	0.0843
**Sleep disorder**	AD	0.93 (0.86~1.02)	0.1134
	Urticaria	1.18 (1.11~1.26)	<0.0001
Psoriasis	1.29 (1.14~1.47)	<0.0001

**Notes:** Adjusted by age, gender, economic status, severity of atopic dermatitis, AD history, concomittent allergic disease. †Odds ratio calculated vs control group with non-AD eczema.

### Difference in mental illness according to AD severity

As the severity of AD increased, the odds of all mental illnesses increased (Table 4). The odds ratio of depression in moderated AD was 1.75 (95% CI=1.42–2.16), and in severe AD, it was 3.15 (95% CI=2.83–3.51). As the severity of AD increased, the odds of anxiety (moderate AD OR=1.59; severe AD OR=2.64) and sleep disorder (mild AD OR=0.88; moderate AD OR=1.31; severe AD OR=2.56) also increased. The prevalence of suicidal ideation (OR=2.68, 95% CI=2.08–3.46) and schizophrenia (OR=3.26; 95% CI=2.40–4.43) was higher in patients with severe AD.

**Table 4:** Risk of mental illnesses according to atopic dermatitis (AD) severity

Mental illness	AD severity	Odds (95% CI)^†^	*P*-value
**Attention-deficit/hyperactivity disorder**	Mild	1.14 (0.84~1.56)	0.3909
	Moderate	1.23 (0.66~2.28)	0.5108
Severe^a^	3.22 (2.36~4.40)	<0.0001
**Autism spectrum disorder**	Mild	1.07 (0.66~1.74)	0.7872
	Moderate	0.96 (0.33~2.84)	0.9438
Severe^a^	2.54 (1.54~4.21)	0.0003
**Conduct disorder**	Mild	0.82 (0.23~2.89)	0.7527
	Moderate	<0.001 (<0.001~ >999.999)	0.9809
Severe	4.32 (1.23~15.10)	0.0221
**Depression**	Mild	0.92 (0.82~1.03)	0.1515
	Moderate^a^	1.75 (1.42~2.16)	<0.0001
Severe^a^	3.15 (2.83~3.51)	<0.0001
**Anxiety**	Mild	0.93 (0.84~1.02)	0.1190
	Moderate^a^	1.59 (1.32~1.91)	<0.0001
Severe^a^	2.64 (2.41~2.88)	<0.0001
**Suicidal ideation**	Mild	0.98 (0.75~1.28)	0.8558
	Moderate	1.22 (0.70~2.14)	0.4877
Severe^a^	2.68 (2.08~3.46)	<0.0001
**Schizophrenia**	Mild	0.81 (0.59~1.12)	0.2081
	Moderate	0.73 (0.33~1.62)	0.4367
Severe^a^	3.26 (2.40~4.43)	<0.0001
**Sleep disorder**	Mild^a^	0.88 (0.78~0.98)	0.0237
	Moderate^a^	1.31 (1.03~1.67)	0.0267
Severe^a^	2.56 (2.30~2.85)	<0.0001

**Notes:** *Adjusted by age, gender, economic status, AD history, concomittent allergic disease. †Odds ratio calculated vs non-AD controls. ^a^Means are significantly different.

### Difference in mental illness according to AD history

As shown in Table 5, patients with AD history had a higher prevalence of depression (OR=1.23; 95% CI=1.06–1.44) and ADHD (OR=3.56; 95% CI=2.21–5.73).

**Table 5:** Risk of mental illnesses according to atopic dermatitis (AD) history in AD patients

Mental illness	Past AD history	
Odds (95% CI)^†^	*P*-value
**Attention-deficit/hyperactivity disorder^a^**	3.56 (2.21~5.73)	<0.0001
**Autism spectrum disorder**	1.55 (0.87~2.74)	0.1351
**Conduct disorder**	2.06 (0.46~9.22)	0.3437
**Depression^a^**	1.23 (1.06~1.44)	0.0072
**Anxiety**	1.12 (0.99~1.28)	0.0797
**Suicidal ideation**	0.82 (0.59~1.14)	0.2293
**Schizophrenia**	0.74 (0.50~1.08)	0.1195
**Sleep disorder**	1.07 (0.91~1.25)	0.4133

**Notes:** ^a^Adjusted by age, gender, economic status, severity of atopic dermatitis, concomittent allergic diseases: Means are significantly different. _†_Odds ratio calculated vs control group with no history of atopic dermatitis.

### Difference in mental illness according to concomitant AD

AD patients with allergic conjunctivitis (AC) showed a higher prevalence of ADHD (OR=1.53; 95% CI=1.13–2.07), and conduct disorder (OR=3.02; 95% CI=1.07–8.49) than AD patients without AC. Patients with AD and allergic rhinitis (AR) showed increased anxiety (OR=1.15, 95% CI=1.02–1.30) and sleep disorder (OR=1.31; 95% CI=1.14–1.52) compared to AD patients without AR (Table 6).

**Table 6:** Risk of mental illnesses according to concomitant atopic disease in atopic dermatitis (AD) patients

Mental illness	Allergic conjunctivitis		Allergic rhinitis		Asthma	
Odds (95% CI)	*P*-value	Odds (95% CI)	*P*-value	Odds (95% CI)	*P*-value
**Attention-deficit/hyperactivity disorder**	1.53 (1.13~2.07)	0.0063	1.21 (0.91~1.60)	0.2009	0.44 (0.30~0.65)	<0.0001
**Autism spectrum disorder**	1.14 (0.66~1.99)	0.6383	0.75 (0.47~1.20)	0.2280	0.54 (0.30~1.00)	0.0502
**Conduct disorder**	3.02 (1.07~8.49)	0.0363	0.38 (0.13~1.12)	0.0796	1.13 (0.32~4.01)	0.8546
**Depression**	1.00 (0.83~1.20)	0.9943	0.92 (0.80~1.05)	0.2272	0.67 (0.55~0.83)	0.0002
**Anxiety**	1.12 (0.95~1.31)	0.1729	1.15 (1.02~1.30)	0.0187	0.97 (0.82~1.14)	0.6732
**Suicidal ideation**	0.70 (0.43~1.15)	0.1571	1.10 (0.80~1.52)	0.5497	1.03 (0.66~1.59)	0.9124
**Schizophrenia**	0.74 (0.42~1.31)	0.3031	0.63 (0.43~0.93)	0.0188	0.50 (0.26~0.95)	0.0355
**Sleep disorder**	1.10 (0.91~1.34)	0.3331	1.31 (1.14~1.52)	0.0002	0.92 (0.75~1.13)	0.4021

**Notes:** Adjusted by age, gender, economic status, severity of atopic dermatitis, AD history, †Odds ratio calculated vs. control group with non-AD eczema.

## Discussion

Our study investigated the associations between AD and several mental illnesses across all age groups: children, adults, and the elderly. Chronic skin disorders such as atopic eczema, psoriasis, and chronic urticaria can significantly influence psychological distress. Furthermore, AD is a well-known chronic eczema and is often associated with other atopic disorders, such as AC, AR, and asthma, and major depression is known to be one of the main disorders associated with chronic skin disorders. A psychiatric epidemiological survey by the Ministry of Health and Welfare found that the lifetime prevalence rate of Korean MDD was 5%, and the period prevalence rate was 1.5% of the total Korean population in 2016.^10^ We calculated the period prevalence of depression in AD patients to be 2.47%, which was higher than the overall Korean rate.

There have been a few epidemiological studies on relationships between AD and psychological distress in children and adults. Some studies reported that patients with AD have more marked depression than healthy adults, and that patients with more severe AD are more depressed.^11^,^12^ Like AD, chronic allergic contact dermatitis affects behavioral and physiological stress.^13^ Seborrheic dermatitis is more prevalent in patients with depression,^14^ and psoriasis and urticaria patients are more likely to have depression.^15^,^16^ Most of these large-population based studies analyzed populations in comparison to a normal control group. We compared prevalence between patients with AD and non-AD, such as other chronic dermatologic disease (eg, seborrheic dermatitis, irritant or allergic contact dermatitis, urticaria, and psoriasis). Our results showed the prevalence of depression was not significantly different between AD and non-AD patients (Table 2). However, more severe AD showed a higher odds ratio for depression (Table 4). In addition, patients who had a history of AD had prevalence of depression (Table 5). History of AD could be interpreted as long-term AD as well as an AD history in infancy or childhood. We suggested that the severity and duration of dermatitis contribute to depression as well as the type of dermatitis.

It has been suggested that some dermatologic patients’ depressive symptoms are associated with cosmetic disfigurement and body image problems, and facial involvement has been associated with depressive mood.^17^,^18^ A prior study examining the relationship between pruritus and depression among a group of patients with pruritic skin disorders, including psoriasis, AD, and chronic idiopathic urticaria, found that patients with higher pruritic scores also had higher depression scores.^19^

We conducted a logistic regression analysis of AD, urticaria, and psoriasis separately, with nonatopic eczema as the reference group (Table 3). As a result, although the odds ratio was not high, the prevalence of depression in patients with psoriasis was significantly higher. Gupta and Gupta^17^ examined the prevalence of depression (measured by the Carroll Rating Scale for Depression) among 480 patients, and found that psoriasis and acne were associated with higher depression scores than alopecia areata and AD. Patients with both urticaria and psoriasis had higher prevalence of anxiety and sleep disorder. There was no significant difference in anxiety and sleep disorder prevalence between patients with AD and nonatopic eczema. However, severe AD was associated with increased prevalence of both anxiety and sleep disorder (Table 4).

Previous studies on the link between anxiety and AD are controversial. Several studies have reported no significant difference in anxiety levels between AD patients and normal controls,^12^ and no significant correlation between AD severity and anxiety.^20^ However, other studies have demonstrated that patients with AD are more likely to have anxiety than healthy individuals.^5^,^6^ In addition, a recent large population-based study showed that moderate-to-severe AD was significantly associated with an increased risk of anxiolytic (HR=1.66; 95% CI=1.56–1.77) and antidepressant drug use (HR=1.24; 95% CI=1.16–1.31), while patients with mild AD only had a slightly increased risk of anxiolytic drug use (HR=1.08; 95% CI=1.01–1.16).^21^

Previous studies have demonstrated that children in the US with AD have higher prevalence of ADHD, autism, and conduct disorder than unaffected peers.^5^ Our results showed that AD patients have higher prevalence of ADHD, ASD, and conduct disorder than non-AD patients. These mental illnesses could have more specific implications, such as a genetic relationship with AD.

The mechanisms underlying mental illnesses and AD are unknown. One of the theoretical mechanisms explaining the association between mental illness and AD is the chronic nature of the skin disease. Mental illnesses could be a common end result of many chronic disorders, including AD. Blackman et al^22^ found that children with any chronic illness had an increased risk of emotional and behavioral problems, including ADHD. However, in our results, the prevalence rates of ADHD, ASD, and conduct disorder were significantly higher in AD than in other chronic skin diseases. Studies have observed increased levels of pro-inflammatory cytokines in AD, and increased cytokines could lead to depression, anxiety, and autism.^23^,^24^ Schmitt et al^25^ suggested that the sustained overexpression of inflammatory mediators released during atopic responses could affect the brain circuits associated with ADHD, making children vulnerable to ADHD symptoms. Both AD and ADHD have complex genetic susceptibility and environmental factors that precipitate into disease manifestation. Several candidate genes encoding major elements of the immune system and proteins involved in the regulation of Th1/Th2 cell differentiation and effector function for atopic traits have been identified.^26^ In particular, genetic variants in the gene encoding signal transducer and activator of transcription 6 (Stat6), a key regulatory element of Th2 immune response, have been associated with atopy-related traits.^27^,^28^ Stat6 is also highly expressed in the central nervous system and is suggested to play a major role in ADHD pathogenesis.^29^ In addition, previous research on the association between atopy and behavioral symptoms in twins has supported the hypothesis of shared genetic factors influencing the risk for atopic and behavioral disorders.^30^

We investigated whether AC, AR, or asthma increased mental illnesses in AD patients. The results showed that AC was more associated with the prevalence of ADHD and conduct disorder than AD alone. AR was associated with an increased prevalence of anxiety and sleep disorder. However, asthma was associated with lower prevalence of ADHD and depression. Prior studies have suggested a link between allergies and ADHD. A meta-analysis showed a stronger association between children with ADHD and asthma than control groups. For AR, AD, and AC, the odds were slightly higher in children with ADHD than in children without ADHD.^31^ Cheng et al^6^ revealed that AR associated with AD resulted in a higher risk of depressive disorders and anxiety disorders than in the absence of AR. Further studies may be needed to explain the comorbidity of atopic disease and mental illnesses.

In conclusion, although the prevalence of depression was higher in patients with AD than in the overall Korean population, there was no significant difference in the prevalence between AD and non-AD patients. However, the severity and history of AD were closely related to the prevalence of depression. The presence of AD was associated with higher prevalence of ADHD, ASD, and conduct disorder than nonatopic dermatologic disease. Further studies are required to investigate the underlying disease mechanisms between AD and these mental illnesses.

## Approval

The Institutional Review Board of the Kyung Hee University Hospital has approved the study (KMC IRB 2016-05-406).
